# Impact of Polytrauma and Acute Respiratory Distress Syndrome on Markers of Fibrinolysis: A Prospective Pilot Study

**DOI:** 10.3389/fmed.2020.00194

**Published:** 2020-06-02

**Authors:** Lukas L. Negrin, Michel Dedeyan, Stefan Plesser, Stefan Hajdu

**Affiliations:** Department of Orthopedics and Trauma Surgery, Medical University of Vienna, Vienna, Austria

**Keywords:** acute respiratory distress syndrome, ARDS, biomarker, fibrinolysis, plasminogen activator inhibitor type-1, pneumonia, polytraumatized patients, tissue plasminogen activator

## Abstract

Acute respiratory distress syndrome (ARDS), which is associated with major morbidity and high mortality, is commonly developed by polytraumatized patients. Its pathogenesis is complex, and its development is difficult to anticipate, as candidate biomarkers for the prediction of ARDS were found not to be reliable for clinical use. In this prospective study, we assessed the serum antigen levels of tissue plasminogen activator (tPA) and plasminogen activator inhibitor type-1 (PAI-1) of 28 survivors of blunt polytrauma (age ≥18 years; injury severity score ≥16) at admission and on days 1, 3, 5, 7, 10, 14, and 21 of hospitalization. Our results show that these patients presented high mean tPA and PAI-1 antigen levels at admission; despite their decline, these parameters remained elevated for 3 weeks. Over this period, the mean tPA antigen level was higher in polytrauma victims suffering from ARDS than in those without ARDS, whereas the mean PAI-1 level was higher in polytrauma victims sustaining pneumonia than in those without pneumonia. Moreover, in each individual developing ARDS, the polytrauma-related elevated tPA antigen level either continued to rise after admission or suffered a second increase up to the onset of ARDS, declining immediately thereafter. Therefore, our findings support the assessment of serum tPA antigen levels after the initial treatment of polytraumatized patients, as this parameter shows potential as a biomarker for the development of ARDS and for the consequent identification of high-risk individuals.

## Introduction

Acute respiratory distress syndrome (ARDS) is a common complication in polytrauma victims, particularly those with chest injuries, and a major cause of mortality and morbidity. Many survivors experience cognitive and functional impairment, psychiatric sequelae, and diminished quality of life (1, 2). ARDS is a highly life-threatening, clinically defined heterogeneous condition regarding both etiology and clinical course that is triggered by either a direct insult (such as pneumonia, lung contusion, aspiration of gastric contents, and inhalation injury) or an indirect insult (such as sepsis, transfusion of blood products, acute pancreatitis, and trauma to regions other than the chest) to the lung (3, 4), causing epithelial and endothelial injury (5). According to the final version of the Berlin definition, ARDS is considered a syndrome of acute onset, with bilateral opacities on chest imaging and noncardiogenic respiratory failure, leading to oxygenation impairment (6). A variety of cellular and molecular mechanisms contribute to the complex pathophysiology of ARDS, including inflammation-induced coagulation and reduced fibrinolysis (7), which favor excessive intraalveolar fibrin deposition (8).

Fibrinolysis is activated by the serine protease tissue plasminogen activator (tPA), which is secreted into the plasma as an active single-chain enzyme (9) primarily by vascular endothelial cells through two pathways: consecutive secretion, in which proteins are continuously released as fast as they are synthesized, resulting in a low steady output and stable level of active tPA (10); and regulated secretion, in which newly synthesized tPA is stored at high concentrations in organelles and secreted only in response to an appropriate stimulus (11). After vascular injury, a rapid, short-term release of tPA occurs within minutes (12). When a fibrin clot forms on the wall of an injured vessel, tPA binds to fibrin and catalyzes the conversion of the circulating zymogen plasminogen to the active serine protease plasmin (13), which proteolytically degrades the fibrin clot (14). Once plasmin is generated, it cleaves the native single-chain tPA and transforms it into more active two-chain polypeptides (15).

To maintain homeostasis between thrombus formation and fibrinolysis plasminogen activator inhibitor type-1 (PAI-1) (16), the primary physiological inhibitor of tPA (17), PAI-1, and tPA are simultaneously released (18). Plasma and platelets represent two distinct pools of PAI-1 (19). While endothelial cells and hepatocytes are thought to be the major sources of plasma PAI-1 (20), megakaryocytes are known to synthesize PAI-1 (21, 22) that is stored at high concentrations in platelet α-granules (17, 23). The tPA inhibitor is released into the plasma when platelets degranulate and accumulate in the growing thrombus after vascular injury (16). PAI-1 is synthesized and secreted in an active form that is spontaneously converted to its latent (inactive) form (24). Active PAI-1 can rapidly inhibit both active single- and two-chain tPA by forming an inactive 1:1 complex (25), thus stabilizing fibrin and promoting the formation of lysis-resistant fibrin clots (26).

The tPA antigen represents the total plasma tPA content, thus comprising active and inactive tPA in complex with various inhibitors, mainly PAI-1. PAI-1 antigen, however, is composed of active, latent, and complex-bound PAI-1. Since plasma PAI-1 exists in molar excess relative to tPA (27), only a small fraction of tPA antigen circulates as an active enzyme (28). When the level of active PAI-1 increases in plasma, more active tPA is inhibited, leading to a lower level of active tPA and higher level of the tPA/PAI-1 complex. Since the complex is cleared slower than active tPA (28), the clearance of total tPA antigen is effectively slowed down, resulting in a higher level of plasma tPA antigen.

Several biomarker candidates have already been investigated in various patient populations regarding early identification and predicting the prognosis of ARDS in high-risk individuals; unfortunately, they were not considered reliable for clinical use (4, 29). Interestingly, Ozolina et al. (30) assessed the plasma concentrations of coagulation and fibrinolysis markers (tissue factor, tPA, and PAI-1) in ARDS and non-ARDS patients of mixed etiologies at enrollment and at the third and seventh days of intensive care. They found increased levels of tissue factor and PAI-1 antigen at the seventh day in patients diagnosed with ARDS, indicating a supportive diagnostic role for these biomarkers. tPA antigen concentration, however, did not change significantly during the observation time.

Since both increased PAI-1 (31) and tPA antigen (32) levels are thought to reflect endothelial damage, we assumed that serum tPA and PAI-1 antigen levels would significantly increase after polytrauma and would remain elevated over a specific period. Moreover, we expected a further increase in the serum tPA and PAI-1 antigen levels caused by an additional release from highly damaged pulmonary endothelial cells in individuals developing ARDS. Thus, we hypothesized that tPA and PAI-1 antigen levels—or their changes during hospitalization—may identify polytraumatized patients at risk of developing ARDS and eventually pneumonia.

The objectives of this study were to compare the individual and mean time courses of tPA and PAI-1 differences in curve progression between subgroups that combine patients developing/not developing ARDS and sustaining/not sustaining pneumonia in order to determine their predictive value for the development of these complications.

## Patients and Methods

### Patients

Our prospective study enrolled 28 consecutive blunt polytrauma survivors, who were directly admitted to our level I trauma center within 1 year and transferred to the intensive care unit after initial treatment. Inclusion criteria were as follows: (1) minimum age of 18 years, (2) Injury Severity Score (ISS) ≥ 16, (3) no anticoagulant medication before trauma, and (4) no tranexamic acid treatment to avoid hyperfibrinolysis. Burn victims and patients with known history of malignancies, asthma (stages 3/4), pulmonary fibrosis, pneumonitis, chronic obstructive pulmonary disease (COPD) Gold III/IV, autoimmune disorders, previous organ transplantation, or ongoing immunosuppressive therapy were excluded.

### Study Protocol

According to in-hospital guidelines, once mechanical ventilation was considered necessary for any polytraumatized patient, the same lung protective protocol based on volume assist-control ventilation, including low tidal volume (6 mL/kg predicted body weight), plateau pressure limit [<30 cm H_2_O, adequate high PEEP (5–24 cm H_2_O)], and balanced respiratory rate (6–35 breaths/min), was applied. Ten healthy volunteer adults were included in our control group, and only one blood sample was taken from them. ARDS was diagnosed according to the Berlin definition (33). Pneumonia was identified by abnormal temperature (>38°C or <35.5°C); either leukocytosis (white cell count > 10 000/mm^3^ or > 10% immature forms) or leucopenia (white cell count <4000/mm^3^); macroscopically purulent sputum; and presence of new cough, dyspnea, and/or tachypnea (in the case of spontaneous breathing patients), as well as by new or changing infiltrate on the chest radiograph.

### Biomarker Assessment

During the initial examination at admission (day 0) and on days 1, 3, 5, 7, 10, 14, and 21 of hospitalization, one separation gel tube (Vacuette^®^ 8 mL; Greiner Bio-One International) was used for each polytraumatized patient for biomarker level measurement as long as he or she consented. Immediately after sampling, blood was centrifuged at 3 000 × g for 15 min at room temperature, and serum was collected and stored at −80°C until assayed. For biomarker analyses, we employed Luminex multianalyte technology. Together with the ProcartaPlex Basic Kit EPX010-10420-901, the “Human tPA Simplex” and “Human PAI-1 Simplex” kits were used to measure tPA and PAI-1 antigen levels, respectively. We performed all measurements in technical duplicates and calculated the respective mean values. The patients were informed about blood sampling at the earliest time point possible. If written consent was not provided, no further blood samples were taken, and the previously sampled material was destroyed at the request of the patient.

### Statistical Analysis

Statistical analysis was performed using the statistical software R 3.5 (34). Demographic data and biomarker levels in the text and tables are presented as mean and range (the latter in square brackets), whereas tPA and PAI-1 antigen levels are displayed as mean ± standard error of the mean (SEM) in graphics for group comparisons. If two SEM bars did not overlap, an independent-samples *t*-test was performed to determine if the means were significantly different at the specific time point. Dependent *t*-tests were conducted to compare paired samples. To test for equality of the mean time trajectories, a linear mixed effects model was fit. The model included time as categorical predictor as well as group and the interaction between time and group. A random effect for the factor patient was included to account for the dependency of observations made in the same patient at different time points. Error variances were modeled as heterogeneous between different time points. A Wald test was used to test the null hypothesis, i.e., the mean difference between the two groups is zero at all investigated time points. Regarding demographic data, the Mann–Whitney *U* test was applied to compare continuous variables, whereas categorical data were analyzed by means of the chi-square test. The Pearson correlation coefficient *r* was calculated to test the association between selected parameters. In general, *p* < 0.05 was considered significant. Intergroup differences in biomarker levels not reaching statistical significance are indicated by NS (not significant) without specifying the *p*-value.

## Results

### Clinical Course

Twenty-one males and seven females were enrolled in our study. Demographic data are presented in Table 1. The causes of polytrauma were car crashes (11); vehicle-, streetcar-, or train-pedestrian collisions (9); or falls from a height ≥2 m (6), as well as overrun by a wheel loader (1) and raid (1). ARDS was diagnosed in 11 individuals and pneumonia in 12. Patients who developed pneumonia clearly showed distinct signs not earlier than day 4 and not later than day 10 after admission. ARDS developed first in each of the six individuals suffering from both ARDS and pneumonia. Secondary surgery was necessary in nine patients and included soft tissue revision, definitive fracture fixation, laparotomy, hematoma evacuation, and tracheotomy. The clinical parameters assessed in our study group are displayed in Table 2. Of these parameters, tPA antigen levels at day 0 solely correlated with the base excess at admission (*r* = −0.575; *p* = 0.001), whereas no correlations were detected for PAI-1 antigen levels at day 0. Noteworthy, correlation coefficients of 0.335 (*p* = 0.081) and −0.073 (*p* = 0.712) were calculated between the ISS value and tPA antigen levels at day 0 and between the ISS value and PAI-1 antigen levels at day 0, respectively.

**Table 1:** Demographic data.

	**Total**	**ARDS**	**Non-ARDS**	***p*-value**
Number of patients (*n*)	28	11	17	
Age (years)	38.4 [18–85]	29.7 [18–48]	44.0 [18–85]	0.134
ISS	35.1 [21–50]	37.4 [29–50]	33.7 [21–50]	0.264
AIS_Thorax_≥3 (*n*)	18 (64.3%)	8 (72.7%)	10 (58.8%)	0.453
AIS_Head_ ≥ 3 (*n*)	14 (50%)	4 (36.4%)	10 (58.8%)	0.246
ICU (days)	22.3 [8–49]	28.8 (16–49]	18.1 [8–42]	0.004
LOS (days)	45.5 [9–97]	61.7 [21–97]	34.9 [9–62]	0.017
Ventilation (days)	13.6 [1–42]	18.9 [8–42]	10.2 [1–31]	0.017
Lung contusion (*n*)	24 (85.7%)	10 (90.9%)	14 (82.4%)	0.572
Pneumonia (*n*)	12 (42.9%)	6 (54.5%)	6 (35.3%)	0.315
ECMO (*n*)	2 (7.1%)	2 (18.2%)	0	0.068
Secondary surgery (*n*)	9 (32.1%)	6 (54.5%)	3 (17.6%)	0.041

*AIS, Abbreviated Injury Scale; ECMO, extracorporeal membrane oxygenation; ICU, length of stay in the intensive care unit; ISS, Injury Severity Score; LOS, length of stay in the hospital*.

**Table 2:** Clinical parameters at admission.

**Clinical parameter**	**Data (mean and range)**
tPA antigen level at admission	5529 [1013–18759] pg/mL
PAI-1 antigen level at admission	16296 [3194–41063] pg/mL
Lactate at admission	3.3 [0.8–13.7] mmol/L
Base excess at admission	−5.8 [(−19.1) to (−0.4)] mmol/L
Shock index at admission	0.82 [0.48–1.30]
Hemoglobin at admission	11.2 [5.8–15.7] g/dL
Thrombocytes	188.2 [49–295] /nL
Fibrinogen	198.3 [90–431] mg/dl
NT-ProBNP	85.2 [28–137] pg/mL
Creatine kinase	631.4 [123–3246] ng/mL
Activated partial thromboplastin	41 [28–123] s
Thrombin time	20 [13–120] s

*NT-ProBNP, N-terminal proB-type natriuretic peptide; PAI-1, plasminogen activator inhibitor type-1; tPA, tissue plasminogen activator*.

### tPA Antigen Levels in the Study Group

**Figure 1:**
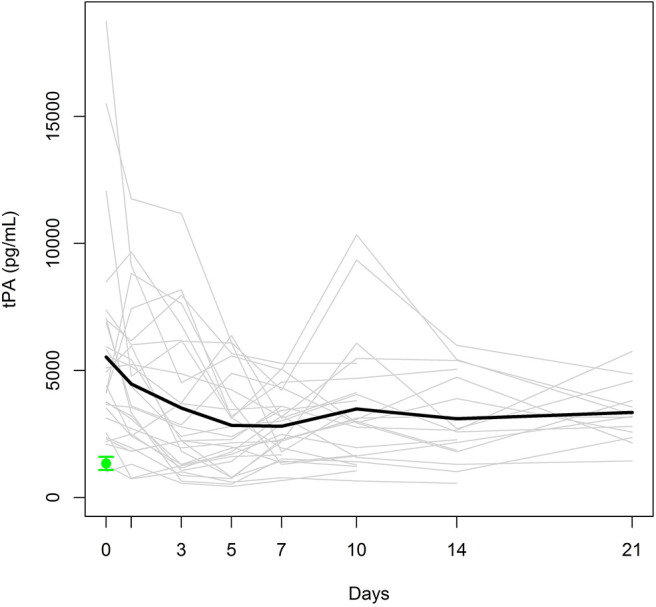
Individual tPA antigen levels (gray lines) and mean tPA antigen level (black bold line) in the study group. Mean ± SEM of tPA in healthy controls (green).

To investigate the natural history of biomarker levels during the first 3 weeks following polytrauma, we analyzed 28 samples up to day 7 (included), whereas only 25, 17, and 13 samples could be analyzed for days 10, 14, and 21, respectively, depending on the date of discharge from the hospital and/or the patient's willingness. Figure 1 displays individual and mean time courses of tPA antigen levels within the first 21 days post-trauma. The polytrauma provoked an instant, sharp increase in the mean tPA antigen level, and the levels at day 0, assessed immediately after admission, were >3-fold higher than the mean tPA antigen level of the healthy control group (1343 [338–2204] pg/mL). After decreasing by 36% from day 0 to day 3 (*p* = 0.004), the mean tPA antigen level basically remained stable for up to 21 days (included).

### Comparison of tPA Antigen Levels in ARDS and Non-ARDS Patients

**Figure 2:**
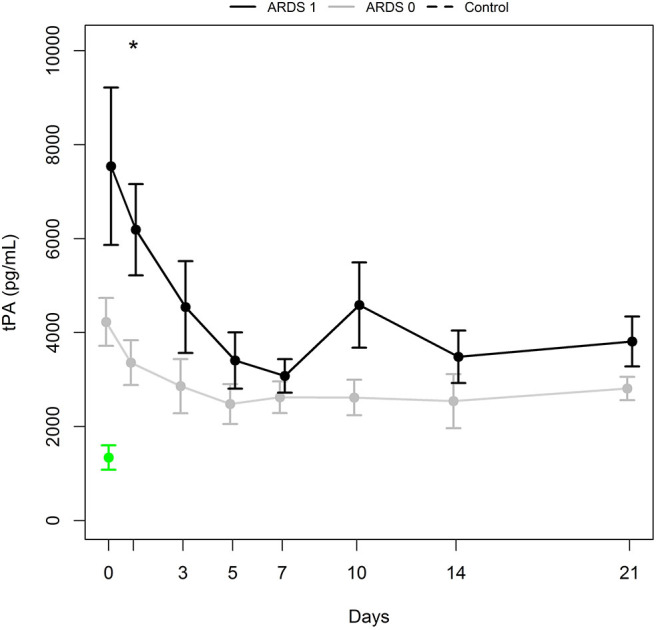
tPA antigen levels (mean ± SEM) in a subgroup of patients with ARDS (ARDS 1, black bold line) and a subgroup with no ARDS (ARDS 0, gray bold line). ^*^Indicates significant difference (*p* = 0.020) between ARDS 1 and ARDS 0 group. Mean ± SEM of tPA in healthy controls (green).

As displayed in Figure 2, the mean tPA antigen level was higher in polytraumatized patients developing ARDS (group ARDS 1) than in those without ARDS (group ARDS 0) for the entire observation period. A significant difference in tPA antigen levels was observed at day 1 (*p* = 0.020), which was confirmed by the Wald test (*p* = 0.004 for the period from day 0 to day 7 and *p* = 0.007 for the period from day 0 to day 21). Furthermore, Wald tests were performed for group comparisons of tPA antigen levels regarding the occurrence of pneumonia, presence of severe thoracic trauma, presence of severe head trauma, and the necessity of secondary surgery (NS).

### Individual tPA Antigen Levels in Patients Developing ARDS

Individual tPA antigen levels and the onset of ARDS (day 0 onset representing an interval of 24 h after the polytrauma occurred) are presented in Table 3. Noteworthy, in each patient who developed ARDS on day 0 or 1, the tPA antigen level increased after polytrauma and did not decrease until the onset of the syndrome. In patients 9 and 10, however, tPA antigen levels considerably increased immediately after trauma, started to decline on day 1, rose again until ARDS developed, and decreased shortly afterwards. Since surgical interventions provoke endothelial damage, we also focused on follow-up procedures in patients sustaining ARDS. Interestingly, the time point of surgery had no impact on the curve progression of individual tPA antigen levels.

**Table 3:** Individual levels of tPA antigen in each polytraumatized patient who developed ARDS.

**Patient**	**ARDS onset**	**Pneu-monia**	**LC**	**tPA levels in pg/mL**							
				**Day 0**	**Day 1**	**Day 3**	**Day 5**	**Day 7**	**Day 10**	**Day 14**	**Day 21**
1	Day 0	Yes	Yes	5,589	**5,171**	3,693	3,461	3,596	2,769	2,644	5,757
2	Day 1	No	Yes	4,157	8,826	**7,641**	3,793	1,305	1,635	2,169	3,196
3	Day 1	No	Yes	2,202	2,499	**1,160**	1,794	3,214	1,575	1,306	1,439
4	Day 0	No	Yes	15,509	**11,760**	11,182	6,104	4,225	9,347	5,992	4,867
5	Day 1	Yes	Yes	4,944	5,972	**1,822**	787	2,204	6,074	2,714	4,587
6	Day 1	Yes	Yes	8,488	9,662	**6,784**	3,191	4,538	4,689	5,051	NA
7	Day 0	Yes	Yes	2,363	**1,960**	865	746	2,295	3,002	1,861	NA
8	Day 0	No	Yes	3,771	**3,095**	1,301	1,702	2,479	1,962	2,277	NA
9	Day 3	Yes	Yes	12,065	4,772	6,154	**6,081**	1,795	3,577	NA	NA
10	Day 10	Yes	No	18,759	9,142	4,518	5,575	5,032	10,349	**5,431**	3,273
11	Day 1	No	Yes	5,111	5,241	**4,871**	4,240	3,177	5,477	5,393	3,565
Mean tPA level				7541	6,191	4,545	3,407	3,078	4,587	3,484	3,812

*ARDS, acute respiratory distress syndrome; LC, lung contusion; tPA, tissue plasminogen activator. NA, not available*.

*Bold numbers highlight the initial decline of tPA level after ARDS developed*.

### PAI-1 Antigen Levels in the Study Group

**Figure 3:**
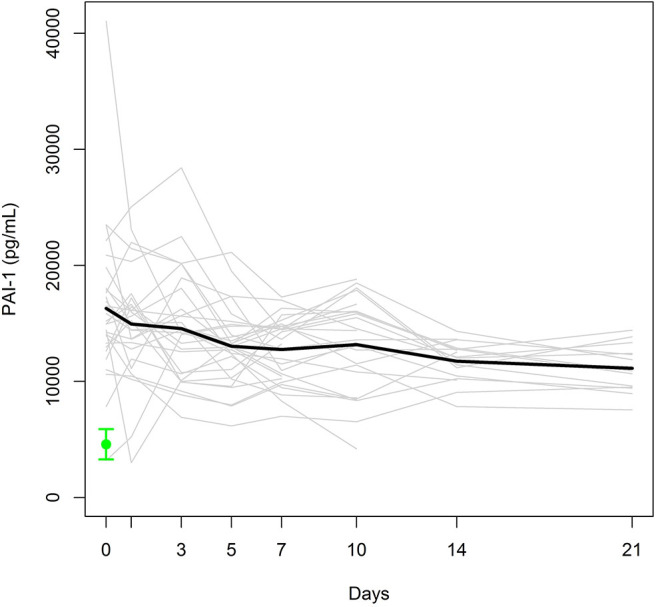
Individual PAI-1 antigen levels (gray lines) and mean PAI-1 antigen level (black bold line) in the study group. Mean ± SEM of PAI-1 in healthy controls (green).

Individual and mean PAI-1 antigen levels are presented in Figure 3. The mean PAI-1 antigen level at day 0, assessed immediately after admission, was 2.6-fold higher than that of healthy controls (4586 [738–9736] pg/mL). Despite the 20% decrease from day 0 to day 5 (*p* = 0.007), the mean PAI-1 antigen level in the study group was higher than the maximum level of the control group during the entire observation period.

### Comparison of PAI-1 Antigen Levels in Pneumonia and Non-pneumonia Patients

**Figure 4:**
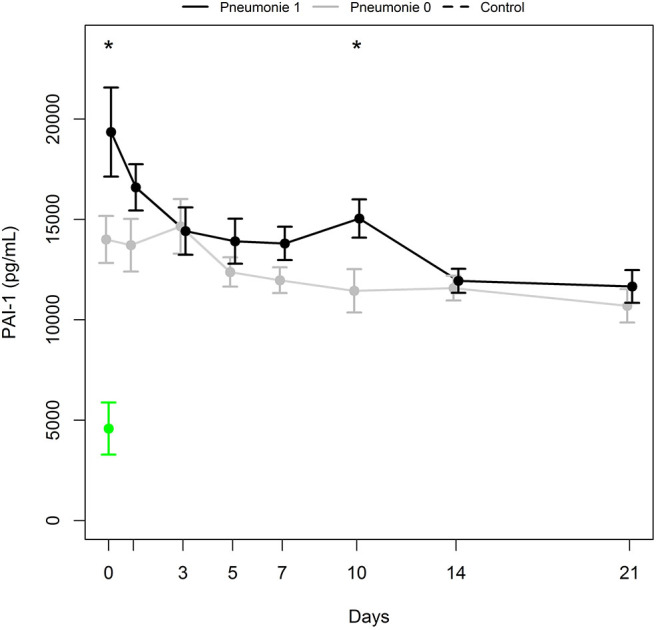
PAI-1 antigen levels (mean ± SEM) in a subgroup of patients with pneumonia (Pneumonia 1, black bold line) and a subgroup with no pneumonia (Pneumonia 0, gray bold line). *Indicates significant difference (*p* ≤ 0.031) between Pneumonia 1 and Pneumonia 0 group. Mean ± SEM of PAI-1 in healthy controls (green).

Differences in mean PAI-1 antigen levels between groups of polytrauma victims developing pneumonia (Pneumonia 1) and not developing pneumonia (Pneumonia 0) were significant at days 0 and 10 (*p* = 0.031 and 0.021, respectively; Figure 4). The Wald test calculated *p* = 0.128 and 0.044 for the first week and first 3 weeks from admission, respectively. PAI-1 antigen levels increased between day 7 and day 10 in 10 of 12 patients developing pneumonia. Interestingly, all 10 patients also suffered from ARDS.

### Correlations Between tPA and PAI-1 Antigen Levels

Finally, the correlation coefficients between tPA and PAI-1 antigen levels, assessed within the first week post-trauma, are presented in Table 4. Also considering the first 2 weeks after the trauma occurred, each pair of tPA antigen levels was significantly correlated, whereas no correlations were found for the tPA antigen levels at day 21. However, the correlation of individual PAI-1 antigen levels over time was significant only in some cases.

**Table 4:** Matrix displaying correlations between antigen levels of tPA and (PAI-1).

	**tPA**	**tPA**	**tPA**	**tPA**	**tPA**	**PAI-1**	**PAI-1**	**PAI-1**	**PAI-1**	**PAI-1**
	**day 0**	**day 1**	**day 3**	**day 5**	**day 7**	**day 0**	**day 1**	**day 3**	**day 5**	**day 7**
tPA day 0	1	0.743^**^	0.582^**^	0.670^**^	0.592^**^	0.624^**^	0.344	0.264	0.188	0.016
tPA day 1	0.743^**^	1	0.840^**^	0.580^**^	0.560^**^	0.411^*^	0.504^**^	0.547^**^	0.238	0.056
tPA day 3	0.582^**^	0.840^**^	1	0.739^**^	0.463^*^	0.290	0.424^*^	0.745^**^	0.417^*^	−0.001
tPA day 5	0.670^**^	0.580^**^	0.739^**^	1	0.598^**^	0.470^*^	0.311	0.532^**^	0.571^**^	0.184
tPA day 7	0.592^**^	0.560^**^	0.463^*^	0.598^**^	1	0.355	0.220	0.086	0.054	0.249
PAI-1 day 0	0.624^**^	0.411^*^	0.290	0.470^*^	0.355	1	0.644^**^	0.401^*^	0.453^*^	0.328
PAI-1 day 1	0.344	0.504^**^	0.424^*^	0.311	0.220	0.644^**^	1	0.580^**^	0.400^*^	0.234
PAI-1 day 3	0.264	0.547^**^	0.745^**^	0.532^**^	0.086	0.401^*^	0.580^**^	1	0.789^**^	0.305
PAI-1 day 5	0.188	0.238	0.417^*^	0.571^**^	0.054	0.453^*^	0.400^*^	0.789^**^	1	0.612^**^
PAI-1 day 7	0.016	0.056	−0.001	0.184	0.249	0.328	0.234	0.305	0.612^**^	1

*PAI-1, plasminogen activator inhibitor-1; tPA, tissue plasminogen activator*.

** *The correlation is significant at the 0.01 level (two tailed)*.

* *The correlation is significant at the 0.05 level (two-tailed)*.

## Discussion

Our data demonstrate that mean tPA and PAI-1 antigen levels significantly increased after polytrauma. Although these levels presented fluctuations, they remained elevated for at least 3 weeks. We speculate that the physical impact at the time of injury and pathophysiological processes hereafter might trigger enhanced consecutive secretion, causing a long-term increase in biomarker levels, whereas regulated secretion might further raise this “steady-state” level for a short period in response to additional endothelial damage.

Furthermore, the strong positive correlation between tPA and PAI-1 antigen levels within the first week post-trauma indicates that tPA and PAI-1 synthesis and their clearance from circulation [by hepatic cells (35)] might be biologically linked. The significant subject correlation over time between each pair of tPA antigen levels within the first 2 weeks post-trauma might be explained by a predominant consecutive secretion, ensuring high continuity in tPA antigen levels. Contrarily, correlation coefficients between PAI-1 antigen levels were significant only in some cases, which might be explained by its regulated secretion from platelets caused by post-trauma triggers, as they vary between individuals.

Our results are not consistent with those obtained by Ozolina et al. (30). Regarding ARDS, mean tPA antigen levels were higher in individuals suffering from this syndrome than in those without ARDS during the entire observation period. Nevertheless, tPA antigen levels at days 0 and 1 were not suitable to predict ARDS, as the levels observed at these days presented high variance due to the different individual injury patterns. Particularly noticeable, however, is the fact that in each polytrauma victim developing ARDS, the tPA antigen level steadily increased or suffered a second increase up to the onset of the syndrome, decreasing immediately thereafter. As each increase in tPA antigen levels during hospitalization may indicate the imminent development of ARDS, successive measurements might allow the identification of high-risk patients in the clinic, and thus enable the timely implementation of effective therapies that can prevent or at least weaken the manifestation of the syndrome. This approach is not only cost- and resource-effective but can also be easily implemented in the clinic by using bedside tests. If included in routine, daily blood sampling and analysis, the assessment of tPA antigen levels would not result in significant additional work and expenses.

The limitations of our study include the fact that we did not perform an *a priori* power analysis to estimate the minimum sample size. Instead, we based our sample size on the number of patients reported in published pilot studies (30, 36–39). Nevertheless, our patient population was too small to perform either subgroup analysis between polytrauma victims suffering from direct and indirect ARDS (diagnosed in 10 patients and 1 patient, respectively) or multivariate regression analysis. Furthermore, blood sampling was determined by the date of discharge from the hospital and/or the patient's willingness, thus resulting in a complete data set of biomarker levels only for the first 7 days post-trauma. Moreover, we could not compare the study group and the healthy control group by means of variance analysis, as we did not sample blood from the healthy controls at the specified time points. Finally, the study population was limited to that admitted at a single level I trauma center.

In conclusion, our pilot study shows an innovative and promising biomarker for ARDS in polytraumatized patients. According to our findings, the increase of serum tPA antigen levels in polytrauma victims after admission should be considered as a warning sign of ARDS development, indicating the potential of plasma tPA antigen, when repeatedly assessed, as a reliable biomarker to identify polytraumatized patients at high risk of developing this syndrome. Hopefully, our results will boost further investigation by large multicenter studies to validate this approach in handling polytrauma victims and eventually other patient groups.

## Data Availability Statement

The datasets generated for this study are available on request to the corresponding author.

## Ethics Statement

The studies involving human participants were reviewed and approved by Ethikkommission der Medizinischen Universität Wien. The patients/participants provided their written informed consent to participate in this study.

## Author Contributions

LN greatly influenced the study design and interpretation of data, drafted the manuscript, and performed the statistical analysis. MD and SP acquired the data, performed blood sampling and analysis, and made critical contributions to the study development. SH contributed to the study design, critically appraised the conclusions drawn from the statistical analysis, revised the manuscript, and supervised the work. All authors read and approved the final manuscript.

## Conflict of Interest

The authors declare that the research was conducted in the absence of any commercial or financial relationships that could be construed as a potential conflict of interest.
